# Local injection of a hexametaphosphate formulation reduces heterotopic ossification *in vivo*

**DOI:** 10.1016/j.mtbio.2020.100059

**Published:** 2020-06-02

**Authors:** T.E. Robinson, N.M. Eisenstein, S.C. Cox, R.J.A. Moakes, A.M. Thompson, Z. Ahmed, E.A.B. Hughes, L.J. Hill, S.A. Stapley, L.M. Grover

**Affiliations:** a Healthcare Technologies Institute, School of Chemical Engineering, University of Birmingham, Edgbaston, B15 2TT, UK; b Royal Centre for Defence Medicine, Birmingham Research Park, Vincent Drive, Edgbaston, B15 2SQ, UK; c Neuroscience and Opthalmology, Institute of Inflammation and Ageing, University of Birmingham, Edgbaston, B15 2TT, UK; d NIHR Surgical Reconstruction and Microbiology Research Centre, Queen Elizabeth Hospital, Edgbaston, B15 2TH, UK; e School of Biomedical Sciences, Institute of Clinical Sciences, University of Birmingham, Edgbaston, B15 2TT, UK

**Keywords:** Ectopic bone, Alginate, Biomaterial, Polyphosphate, Targeted delivery

## Abstract

Heterotopic ossification (HO), the pathological formation of ectopic bone, is a debilitating condition which can cause chronic pain, limit joint movement, and prevent prosthetic limb fitting. The prevalence of this condition has risen in the military population, due to increased survivorship following blast injuries. Current prophylaxes, which aim to target the complex upstream biological pathways, are inconsistently effective ​and have a range of side-effects that make them unsuitable for combat-injured personnel. As such, many patients must undergo further surgery to remove the formed ectopic bone. In this study, a non-toxic, U.S. Food and Drug Administration (FDA) -approved calcium chelator, hexametaphosphate (HMP), is explored as a novel treatment paradigm for this condition, which targets the chemical, rather that biological, ​bone formation pathways. This approach allows not only prevention of pathological bone formation ​but also uniquely facilitates reversal, which current drugs cannot achieve. Targeted, minimally invasive delivery is achieved by loading HMP into an injectable colloidal alginate. These formulations significantly reduce ​the length of the ectopic bone formed in a rodent model of HO, with no effect on the adjacent skeletal bone. This study demonstrates the potential of localized dissolution as a new treatment ​and an alternative to surgery ​for pathological ossification and calcification conditions.

## Graphical abstract

**Figure:**
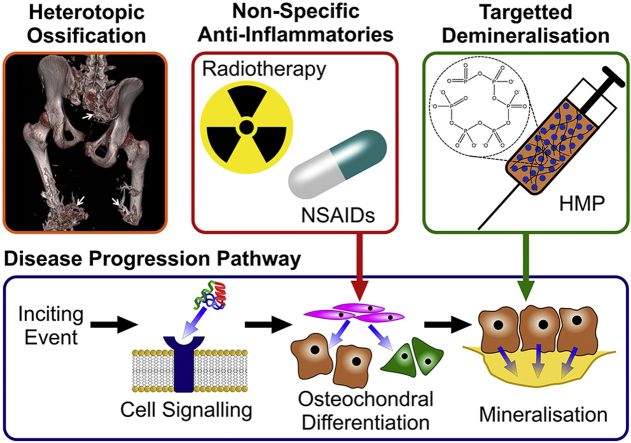

## 1 Introduction

Heterotopic ossification (HO) is the pathological formation of ectopic bone in soft tissues, such as muscle, skin, hypodermis, and fibrous tissue [1]. Complications arising from this condition include chronic pain, skin ulceration, limited movement if formed in joints, and issues with prosthetic limb fitting if it develops in the residual limbs of amputees [2]. HO can be acquired following musculoskeletal trauma, central nervous system injury, and burns ​or may be caused by rare genetic conditions, including fibrodysplasia ossificans progressiva or progressive osseous heteroplasia [3]. Within the military population, HO has become increasingly common because of improved survivorship and the nature of modern combat wounds, particularly the higher incidence of blast injuries. HO is found in 63% of all traumatic or combat-related amputees ​and 80% in those with an amputation through the zone of injury [4]. The increased military occurrence has also highlighted the prevalence of HO in civilian patients, after total hip arthroplasty (24–28% [5]), burn injuries (5.6% [6]), and traumatic brain and spinal cord injuries (4% and 11%, respectively [7]).

**Fig. 1:**
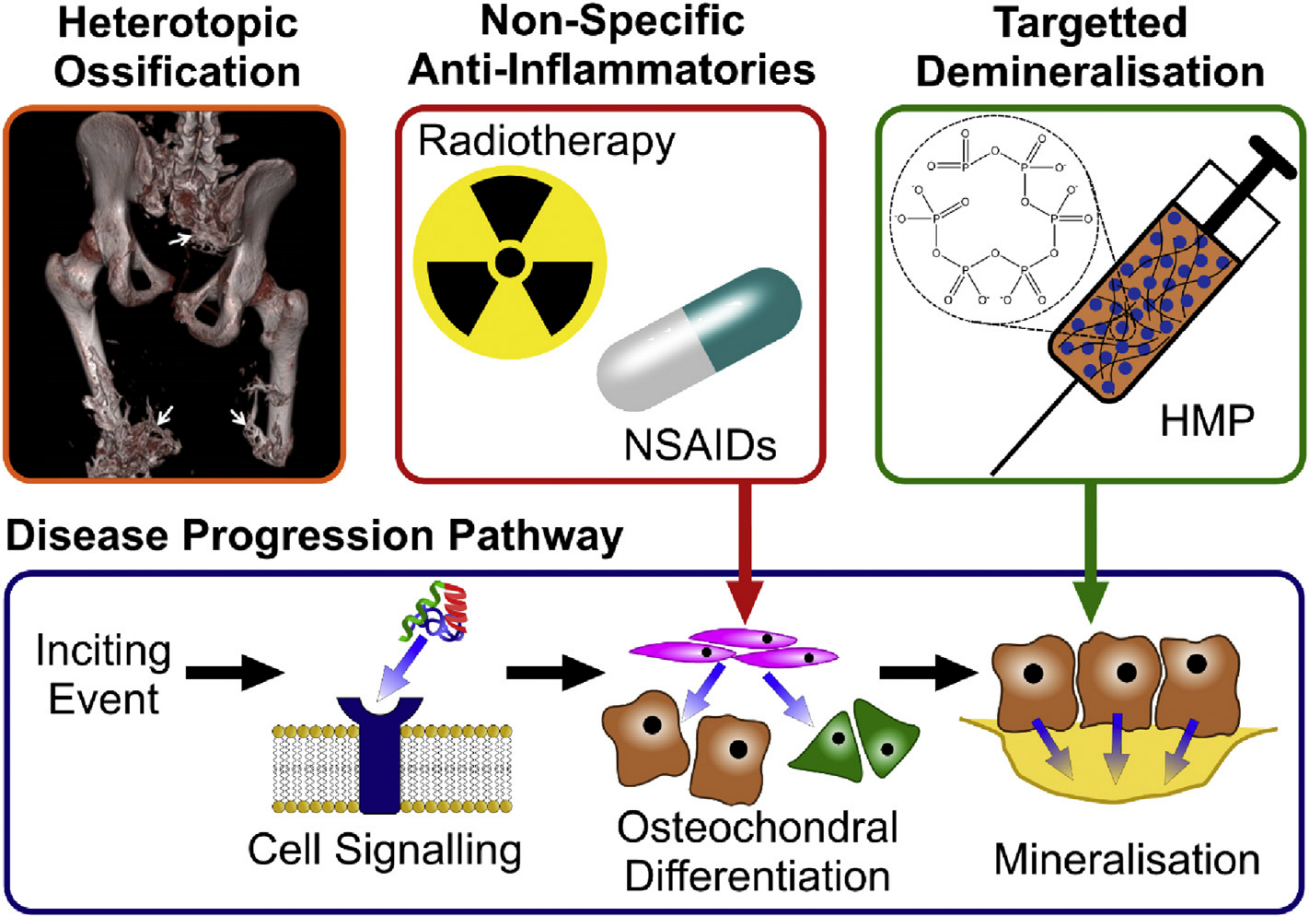
**Schematic showing the progression pathway of HO**. An initial inciting event causes an inflammatory response which recruits progenitor cells to the site. Biochemical signals, released from the cells at the injury site or the recruited inflammatory cells, cause the progenitor cells to differentiate down an osteochondral pathway. Current prophylaxes for HO, radiotherapy and NSAIDs, target this step, aiming to reduce differentiation and proliferation of these bone producing cells. The treatment proposed in this study aims to prevent and reverse mineralization by locally chelating calcium. Image of HO reproduced with permission from John Wiley and Sons [14]. HMP, hexametaphosphate; HO, heterotopic ossification; NSAIDs, non-steroidal antiinflammatory drugs.

Notably, there is a lower prevalence (23%) of HO in civilians after amputation [2]. The large discrepancy in prevalence between military and civilian amputees, as well as the diversity of causes, highlights the complex etiology of HO. There is a general consensus that HO is formed via a combination of systemic factors, such as upregulation of cytokines, and local conditions, including hypoxia, inflammation, and hematoma formation [8]. Kaplan et al. [9] described four factors necessary for the formation of HO: (1) an inciting event, usually traumatic, which may cause a hematoma; (2) cellular signaling from the injured cells ​or the inflammatory cells that move to the site; (3) a supply of undifferentiated mesenchymal stem cells, which will differentiate into chondroblasts and osteoblasts on receiving these signals; and (4) a local environment conducive to HO formation, for example, low pH and hypoxia (Fig. 1). These factors may be applied loosely to all causes of HO; however, given the multiple causes of this disease, there are likely to be several, complex biological signaling pathways that induce osteogenic differentiation. It is speculated that bone morphogenetic proteins ​play a central role, but several other proteins, including transforming growth factor-β, hedgehog, and fibroblast growth factors, are also involved [10]. The potential for multiple complex biological pathways makes designing a treatment strategy for HO difficult, and thus, there are currently no effective pharmacological treatments used clinically [11]. Current treatment options for formed HO are extremely limited. For smaller deposits, range of motion exercises may increase joint mobility; however, these exercises can also exacerbate the condition [12]. Rest and analgesia may help to relieve some symptoms, but the only effective therapy is surgical excision of the formed bone [13]. This comes with the inherent risks of surgery, such as hemorrhage and infection, and no guarantee that the condition will not recur.

The most common prophylaxes currently used to prevent HO are non-steroidal anti-inflammatory drugs (NSAIDs) and radiotherapy. NSAIDs, typically orally administered indomethacin, aim to prevent HO by suppressing proliferation and inducing apoptosis of osteoblasts and chondrocytes [15]. NSAIDs may reduce the incidence of HO ​but can cause gastrointestinal side-effects, acute renal failure, and bleeding, and increase the risk of fracture non-union [[16], [17], [18]]. This can result in discontinuation, even in relatively healthy civilian patients, as their deleterious side-effects become worse than the initial condition. Perioperative radiotherapy is administered to suppress HO by inhibiting mesenchymal stem cell proliferation ​or inducing terminal differentiation [19]. This prevention has been poorly studied, with the exception of HO following hip arthroplasty, with inconclusive timing and dosage guidelines [20,21]. Radiotherapy may also give rise to non-union ​and contracture of soft tissues ​and delay wound healing [22,23]. Both NSAIDs and radiotherapy are non-specific, aiming to reduce HO by decreasing inflammation, and neither are entirely effective. They reduce the probability of developing HO, though effectiveness varies between studies [23]. Furthermore, the vast majority of these studies examine the development of HO after elective joint surgeries in controlled medical environments, where the side-effects may be tolerable. This is magnified for combat-injured patients with complex injuries, in whom delayed wound healing and fracture non-union are unacceptable.

Current research on new prophylactics for HO aims to target the specific biological pathways by which the ectopic bone forms. Strategies such as antibiotic administration, remote adenosine triphosphate hydrolysis, and agonism of retinoic acid receptor gamma have been investigated [[24], [25], [26], [27]]. Despite their potential, the complexity of the biological pathways of HO, which are not completely understood, limits understanding of how these interventions prevent the disease ​and may not prevent all etiologies of HO. Furthermore, therapies which target these upstream biological processes of bone formation, including NSAIDs and radiotherapy, will never be able to treat HO once formed, leaving excision surgery as the only option for many.

The biological mechanisms leading to HO are complex and may have multiple pathways. As such, no specific biological preventions are used clinically, and currently used prophylaxes are non-specific, not entirely effective, and have a range of side-effects. However, all forms of HO share a common final step; formation of solid calcium phosphate mineral in bone. A prevention that targets this chemical pathway would not only be able to treat all forms of this disease ​but would be uniquely able to dissolve formed mineral. This would offer not only a specific prophylactic for HO ​but also a medical alternative to surgery.

Hexametaphosphate (HMP), commonly supplied as a sodium salt, is an inorganic, multivalent polyphosphate. It is used in several industries ​including food, minerals, and ceramics, as well as in medical and dental applications [[28], [29], [30], [31], [32], [33]]. HMP may be used as a deflocculant, by altering the charge of particles, but is more commonly used as a sequestrant ​because it forms strong complexes with metal cations, particularly calcium (Equation (1)).

(1) $${{\left(PO_{3}\right)}_{6}}^{6-}+3Ca^{2+}\to Ca_{3}{\left(PO_{3}\right)}_{6}$$

The early work by Fleisch and Neuman [34] and Fleisch et al. [35] showed that HMP is able to prevent precipitation of calcium phosphate, even in supersaturated solution. HMP has further been shown capable of dissolving solid hydroxyapatite (HA), the main mineral constituent of bone [36,37]. Eisenstein et al. [38] have recently shown that HMP can demineralize biological bone, without affecting the adjacent collagen, and that it is hydrolyzed by alkaline phosphatase, an enzyme present ubiquitously *in vivo*. This is important as it will temporally limit the action of HMP, preventing continuous demineralization. These properties of HMP advocate its use as a therapeutic to target the chemical formation pathway of HO ​to prevent and reverse the mineralization of bone. However, the dissolution mechanism of HMP is not specific to pathological, rather than skeletal, bone. It must therefore be loaded into a delivery vehicle ​for targeted release to the HO site. This study proposes the use of colloidal alginate as a delivery vehicle for HMP. The resulting injectable formulation could be used as a prophylactic or minimally invasive alternative to surgery ​to improve outcomes and quality of life for sufferers of HO. In this manuscript, we report on the formulation of an injectable delivery system and examine its efficacy *in vitro*, *ex vivo*, and in an *in vivo* model of the disease.

## 2 Methods and materials

### 2.1 Study design

The objective of this study was to formulate, optimize, and characterize an injectable HMP-alginate formulation, which may be used as a novel prophylactic and treatment for HO. For *in vitro* evaluation, a standard n ​= ​3 was used for most studies ​and for *in vivo*, n ​= ​6. *In vivo* work was batch randomized, with 3 animals per batch, and blinded ​so that the injector did not know which animals were in the active or control group. As a *de novo* study, time points for injection were based on the stipulations of the project license, and the endpoint was determined by assurance of HO development from initial model validation studies. A moderate level of discomfort at any point was also an endpoint, though this was not enacted in this study. No data were excluded.

### 2.2 Material characterization

#### 2.2.1 Formulation

Alginate-HMP systems were prepared by dissolving HMP powder (sodium hexametaphosphate, general purpose grade, Fisher Scientific) in deionized water ​and then codissolving alginate powder (alginic acid sodium salt from brown algae, BioReagent grade, MW 100–200 ​kDa [39], cn 71238, Sigma-Aldrich), by stirring at 400 ​rpm for 30 ​min at 20 ​°C. No divalent cations were added, such that formulations remained viscous fluids. Where required, pH was adjusted with 0.1 or 1 ​M sodium hydroxide or hydrochloric acid solutions (Sigma-Aldrich).

#### 2.2.2 Rheology

Rheological characterization of formulations was carried out on an AR-G2 rheometer (TA Instruments), using 40 mm diameter sandblasted parallel plates, to minimize wall slip for highly viscous samples, with a 1 mm gap. Shear ramps were carried out by continuously increasing the shear rate from 1 to 1000 s^−1^, over 10 ​min, and measuring the viscosity instantaneously. Recovery behavior was examined by performing a series of peak holds, at a shear rate of 1 s^−1^ and 1000 s^−1^, respectively, for 1 ​min. All measurements were taken at 20 °C.

#### 2.2.3 Zeta potential

Zeta potential of the polymers in solution was determined via electrophoretic light scattering, using a Zetasizer Nano ZS (Malvern), which calculated the zeta potential after measuring the electrophoretic mobility via laser Doppler velocimetry, at a wavelength of 633 ​nm. For each measurement, 10–100 runs were taken at 25 °​C.

#### 2.2.4 Fourier-transform infrared spectroscopy

Scans were performed in transmission mode, with attenuated total reflectance, on a Nicolet 380 Fourier transform infrared (FTIR) spectroscope (Thermo Electron Corporation). For each sample, spectra were averaged from 128 repeats. Samples of HMP and alginate were analyzed directly in their powder form. To prepare the mixed system, a formulation of 1 w/v% alginate and 1 ​M HMP was filtered to collect the precipitate, which was then dried and ground to give a powder for analysis.

#### 2.2.5 Injectability

Injectability studies were carried out on a Z030 universal mechanical tester (Zwick Roell), as shown in Fig. 4A. A displacement-controlled compression test was applied, using a 100 ​N load cell, on the plunger of a 1 ​mL Luer-Lok syringe (Beckton Dickenson), suspended by clamps. The attached needle was either a 19-gauge (690 ​μm internal diameter, 50 ​mm length) or 30-gauge (160 ​μm internal diameter, 12 ​mm length) needle and was initially full. Displacement was carried out over 45 ​mm, and the maximum allowable force was set at 50 ​N, as the limit for a reasonable injection force by hand is 38 ​N [40].

### 2.3 Bioactivity

#### 2.3.1 HMP release

Release from the formulations was studied via the regular dialysis method [41], and conductivity was used to measure concentration. Dialysis tubing, with a nominal molecular weight cutoff of 2000 ​Da, was loaded with 5 ​mL of formulation and tied at each end to create a sealed parcel. Each sample was placed into 50 ​mL of deionized water at 37 ​°C, and the conductivity of the release medium was measured up to two weeks. The HMP concentration in the medium was calculated from the conductivity using a standard curve ​and corrected to remove the contribution to conductivity from alginate, using an alginate only control. The data are presented as a percentage of the total release, with 100% taken as the point at which the HMP concentration is the same both inside and outside the dialysis tubing barrier.

#### *2.3.2* *In vitro* dissolution of HA

HA was synthesized by a sol-gel precipitation method [42]. Discs (12 ​mm diameter, 1 ​mm thickness) were formed in a pellet press and were then sintered at 700 °C for 4 ​h. Pellets were then embedded in EpoFix (Struers) resin ​and were polished with silicon carbide discs (Struers) of decreasing grain size, down to 5 ​μm.

Interferometry was carried out on a MicroXAM2 (Omniscan), using green light. Scans on day 0 were carried out over a depth of 20 ​μm, while scans on day 7 were carried out over a depth of 30 ​μm. All scans had a noise reduction of 0.05. Scanning electron microscopy (SEM) images were taken using secondary electron detection, with a TM3030 Plus (Hitachi) at 15 ​kV. Embedded pellets were attached to a steel mount with carbon tape, sputter coated with 15 ​nm of gold, and connected to the mounting with copper tape ​to improve conductivity. Pellets were analyzed via both modalities, and then, 40 ​μL of 2 w/v% alginate ​with or without 0.2 ​M HMP was applied. The pellets were stored in a closed container with water ​to create a humid environment and reduce evaporation. The formulation was removed with deionized water, the pellets were dried with absorbent paper, taking care not to scratch the surface, and formulation was reapplied in the same location. This was repeated daily, for 5 days, before reanalysis of the pellet surfaces.

#### *2.3.3* *Ex vivo* demineralization of the bone

Femurs were harvested immediately *postmortem* from male Sprague Dawley rats (Charles River) ​and frozen at −20 ​°C until further use. The distal end, with an approximate volume of 30 ​mm^3^, was removed ​and placed in 2 w/v% alginate solution, with or without 0.2 ​M HMP, adjusted to pH 7. Microcomputed tomography (micro-CT) scans were taken at 0, 7, and 14 days with a SkyScan 1172 (Bruker), using the following settings: 0.5 ​mm aluminium filter, current 100 ​mA, voltage 75 ​kV, exposure time 950 ​ms, pixel size 5.4 ​μm, camera resolution 2000 ​× ​1332 pixels, rotation step 0.3°, frame averaging 10. Scans were reconstructed using NRecon (version 1.6.10.2, Bruker), and 3D models were produced in CTVox (version 3.0.0, Bruker). The same scanning, reconstruction, and postreconstruction parameters were used for all scans.

#### *2.3.4* *In vivo* prevention of HO

Ethical approval for this work was given by the University of Birmingham's Animal Welfare and Ethical Review Board, and experiments were licensed by the UK Home Office. All work was carried out in strict accordance to the guidelines of the UK Animal (Scientific Procedures) Act 1986 and the Revised European Directive 1010/63/EU and conformed to the guidelines and recommendation of the use of animals by the Federation of the European Laboratory Animal Science Associations.

Unilateral Achilles tenotomy was performed on the right hind limb of adult male Sprague Dawley rats (Charles River) ​to induce HO [43]. Surgery was performed under general anesthesia, induced and maintained by 5–2% isofluorane, and the incision was closed with two interrupted sutures and sealed with surgical glue. Opioid analgesia, typically buprenorphine, was given as required; NSAIDs were not used at any point in this study.

Surgery and injection of the formulations were performed under sterile conditions, and all equipment used was either purchased sterile ​or autoclaved before use. Formulations were sterilized by passing them through a 0.2 ​μm filter ​and aspirating them into syringes, which were kept sterile until use.

This study was performed in two batches, with 6 rats in each batch: 3 in the treatment group and 3 in the control group. At 2, 4, 6, and 8 weeks after the operation, each animal was given an injection into their right hind limb, directly adjacent to the site of HO formation. The control group was given 200 ​μL of 1 w/v% alginate, and the treatment group was given 200 ​μL of 1 w/v% alginate with 0.2 ​M HMP, via a μm diameter needle. Animals were sacrificed at 10 weeks, by exposure to CO_2_ in rising concentration, and their hind limbs were immediately harvested and frozen at −80 ​°C until scanning.

The tissue was defrosted, and micro-CT scans were taken using a SkyScan 1172 (Bruker). The scan settings used were as follows: 0.5 ​mm aluminium filter, current 100 ​mA, voltage 75 ​kV, exposure time 400 ​ms, pixel size 21.5 ​μm, camera resolution 1000 ​× ​666 pixels, rotation step 0.6°, frame averaging 2. Scans were reconstructed using NRecon (version 1.6.10.2, Bruker), and quantitative analysis was performed in CTAn (version 1.15.4.0, Bruker). For length measurement, the data were first orientated in DataViewer (version 1.5.1.2, Bruker) ​so that the longest axis of the HO was orthogonal to the x-y plane. The data were then loaded in CTAn, and the length was calculated from the height of the z-stack. The cortical bone tissue mineral density (TMD) of the tibia directly adjacent to the HO site (Fig. 6B blue square) was calculated following the method supplied by the manufacturer, by calibrating with phantoms of a known HA density [44]. 3D models were produced in CTVox (version 3.0.0, Bruker). The same scanning, reconstruction, and post reconstruction parameters were used for all scans.

### 2.4 Statistical analysis

Normality was determined using a Shapiro-Wilk test. All data sets were then analyzed using two-tailed unpaired *t*-tests, with *p* ​< ​0.05 defined as significant in all tests (∗, *p* ​< ​0.05; ∗∗, *p* ​< ​0.01; ∗∗∗, *p* ​< ​0.001; ∗∗∗∗, *p* ​< ​0.0001). Standard deviation (SD) was used as the measure of uncertainty. Statistical analysis was performed in Prism (version 7, GraphPad).

## 3 Results

### 3.1 Shear-thinning formulations with recoverable post-shear viscosity

**Fig. 2:**
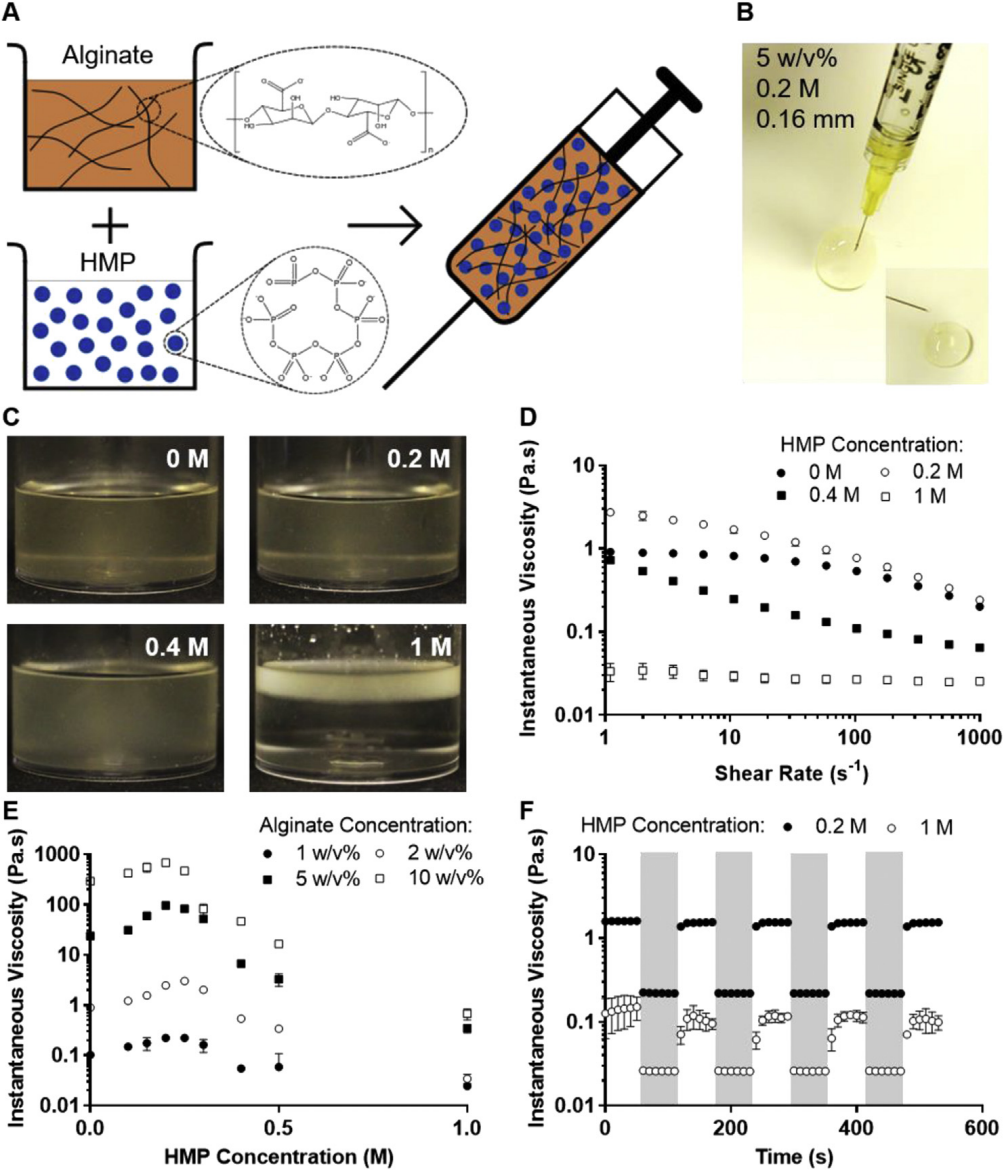
**Formulations are shear-thinning and recover their viscosity following shear**. **(A)** Schematic of the formulation process; HMP and alginate were codissolved in deionized water, creating a formulation which can then be loaded into a syringe. **(B)** Photograph of a formulation (containing 5 w/v% alginate and 0.2 ​M HMP) being injected, with insert demonstrating viscosity recovery after delivery through a 160-μm diameter needle. **(C)** Photographs of 2 w/v% alginate with varying HMP concentrations showing phase separation above 0.2 ​M. **(D)** Shear rate ramps revealing the shear-thinning behavior of 2 w/v% alginate and varying concentrations of HMP. **(E)** Viscosity of formulations at a shear rate of 2 ​s^−1^, as a function of HMP concentration for various alginate concentrations. **(F)** Recovery of viscosity as a function of time, when 2 w/v% alginate was exposed to high shear (1000 ​s^-1^, shaded sections) to mimic injection and then low shear (1 s^−1^, white sections) to assess the ability of the formulation to remain localized. (C), (E), and (F) show mean ​± ​SD (n ​= ​3). SD, standard deviation; HMP, hexametaphosphate.

Therapeutic formulations were prepared by dispersing alginate into an aqueous solution of the HMP (Fig. 2A). This created viscous, fluid formulations which could be injected through a needle ​and subsequently recohere (Fig. 2B). Formulations containing HMP at a concentration of up to 0.2 ​M exhibited a similar yellow-brown color and translucency to alginate alone, however, above this concentration, a precipitate formed and phase separation was observed (Fig. 2C).

Rotational rheology was performed to characterize the shear-dependent viscosity of the formulations. The viscosity of all samples was found to decrease with applied shear, that is, they were shear-thinning (Fig. 2D). HMP concentration was also found to influence the nature of this behavior, demonstrated by the change in concavity of the shear response curves. Furthermore, HMP concentration altered the instantaneous viscosity of the formulations at all tested shear rates. Alginate solution viscosity was increased by raising the HMP concentration, up to a critical value of approximately 0.2 ​M (Fig. 2E). However, further increasing HMP content above this concentration decreased the viscosity. This is concurrent with the observation of precipitate formation at HMP concentrations greater than 0.2 ​M. The time it took for the formulations to recover their standing viscosity following the application of high shear (1000 s^−1^) was also assessed. The 0.2 ​M HMP system, which did not precipitate, recovered its viscosity on the removal of shear almost instantaneously (Fig. 2F). The precipitated samples with 1 ​M HMP, however, displayed thixotropy. There was time dependence to the shear-thinning behavior, with the formulation taking around 30 ​s to recover.

### 3.2 Interaction between the demineralizing agent and polymer network

**Fig. 3:**
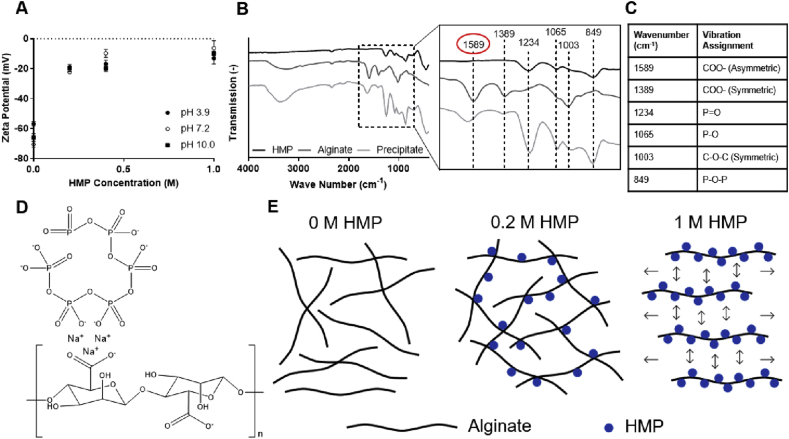
**Molecular interaction between alginate and HMP alters bulk formulation properties**. **(A)** The zeta potential of dilute alginate solutions (0.1 w/v%), at varying pH values, as a function of HMP concentration. **(B)** FTIR spectra of HMP and alginate powders ​and the precipitate formed between 1 w/v% alginate and 1 ​M HMP. Expanded section shows the relevant peaks, with dashed lines highlighting shifts. **(C)** Summary of the chemical bonds assigned to each FTIR peak. **(D)** Schematic of the proposed molecular mechanism by which HMP and alginate interact. **(E)** Illustration of how this interaction causes increased alginate cross-linking at low HMP concentrations ​and precipitation at high HMP concentrations. (A) shows mean ​± ​SD (n ​= ​5) ​and (B) shows the mean of 128 scans. SD, standard deviation; FTIR, Fourier-transform infrared spectroscopy; HMP, hexametaphosphate.

The strong effect of HMP concentration on the rheological properties of the formulations (Fig. 2) suggested that there was a molecular interaction between the alginate and active component. This behavior was found to be concentration dependent, and up to 0.2 ​M HMP, the network density increased, as evidenced by increased viscosity. Above this concentration, precipitation occurred. To investigate the electrostatic forces, the zeta potential of the formulations was measured. Alginate alone in solution is strongly anionic, having a zeta potential more negative than −30 mV, indicating that the electrostatic repulsion between polymer chains is enough to prevent aggregation [45]. However, the addition of HMP, at any concentration tested (0.2, 0.4, and 1 ​M), reduced the zeta potential to less than −30 mV (Fig. 3A). This suggested that it decreased the electrostatic repulsion ​and allowed the polymer chains to come into closer contact, which above 0.2 ​M HMP resulted in flocculation and precipitation. The pH appeared to have little effect on the zeta potential of this system.

To investigate the chemical mechanisms of this interaction FTIR ​was conducted on the individual components, alginate and HMP, and the formed precipitate. No new peaks were seen in the spectra for the precipitate that were not present in the raw material spectra, discounting covalent bonding between species. For some peaks, however, the wavenumber was observed to shift, suggesting ionic interactions between the alginate and HMP (Fig. 3B). In particular, there was a large shift in the peak at 1589 ​cm^−1^ (circled), which corresponded to the COO^−^ group on the alginate [46], and shifts in the peaks at 1234 and 1065 ​cm^−1^, which corresponded to the P=O and P–O groups, respectively, on the HMP (Fig. 3C) [29]. This implied that these groups were interacting ionically, and thus a mechanism can be proposed, whereby the resonating groups on both species stabilize around one or more sodium ions, of which there are an abundance in these formulations (Fig. 3D). This may explain the effects of HMP on the bulk rheological properties. At concentrations up to 0.2 ​M, each HMP ion binds multiple polymer chains, increasing the network density, but without causing phase separation. At higher HMP concentrations, the increased number of interactions causes the polymer chains to come much closer together, which forces the water phase out (Fig. 3E). This polymer-rich phase, having a lower density, ​then creams out, leaving a polymer-poor phase below (Fig. 2C). The precipitate removes the polymer available to form the network, reducing the viscosity of the system.

### 3.3 Injectable formulations for targeted release

**Fig. 4:**
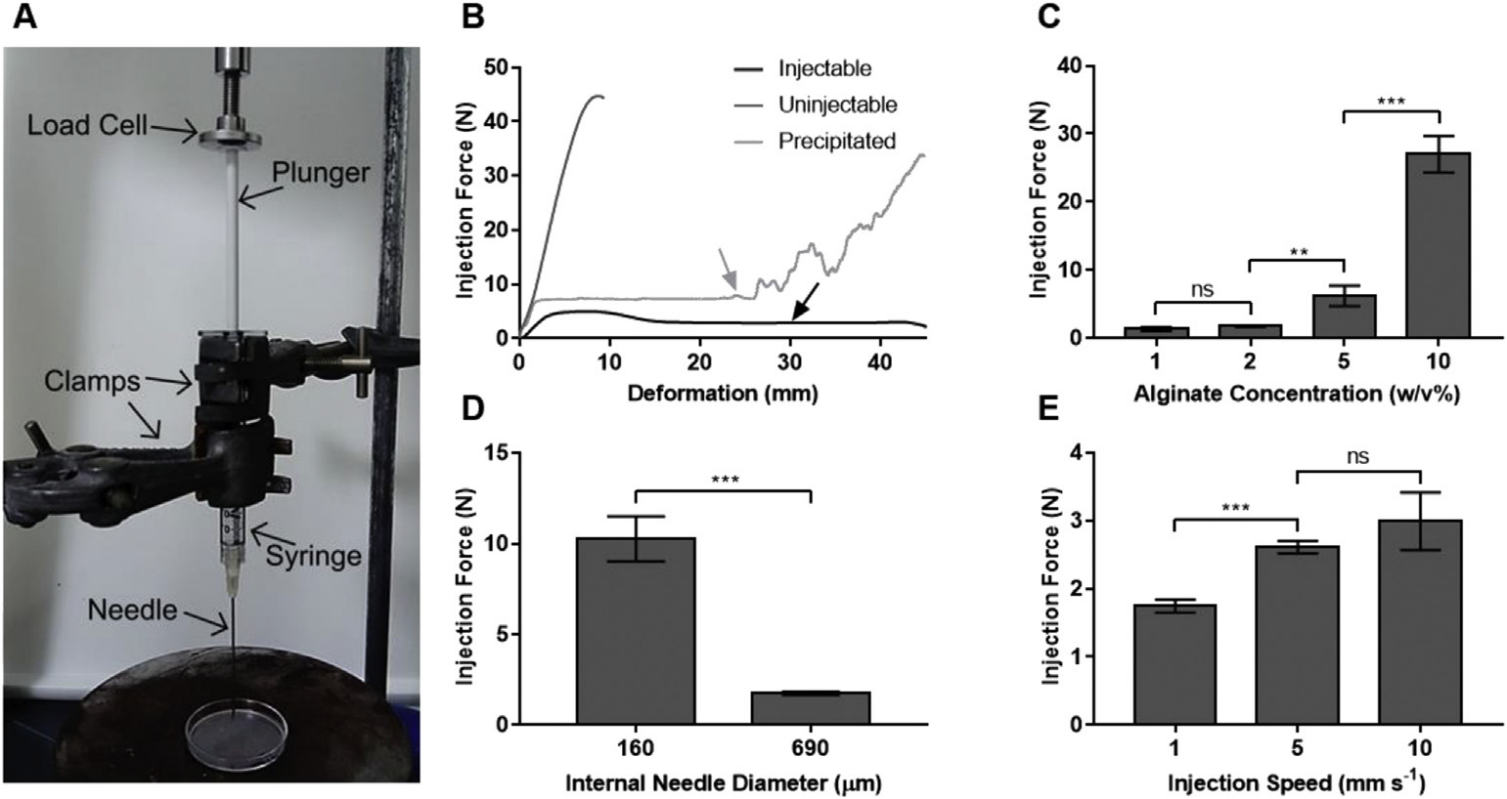
**Formulations are injectable through a hypodermic needle**. **(A)** Annotated photograph showing the setup for quantitative injectability studies; the syringe was suspended by clamps, and the plunger was uniaxially compressed by the cross-head. **(B)** Sample curves from injectability studies. The black line (2 w/v% alginate, 0.2 ​M HMP, 690 ​μm needle diameter, 10 ​mm ​s^−1^ injection speed) shows an injectable sample, and the black arrow shows the plateau on the curve where the value for injection force is taken. The dark gray line (5 w/v% alginate, 0.2 ​M HMP, 160 ​μm needle diameter, 10 ​mm ​s^−1^ injection speed) shows a sample that has reached the imposed limit on injection force and is deemed to be uninjectable. The light gray line (2 w/v% alginate, 1 ​M HMP, 160 ​μm needle diameter, 1 ​mm ​s^−1^ injection speed) shows a sample that has precipitated, and the light gray arrow shows the onset of instability due to filter pressing. **(C)** The force required to inject formulations (0.2 ​M HMP, 690 ​μm needle diameter, 1 ​mm ​s^−1^ injection speed) at varying alginate concentrations. **(D)** The force required to inject formulations (2 w/v% alginate, 0.2 ​M HMP, 1 ​mm ​s^−1^ injection speed) at varying internal needle diameters. **(E)** The force required to inject formulations (2 w/v% alginate, 0.2 ​M HMP, 690 ​μm needle diameter) at varying injection speeds. (C), (D), and (E) show mean ​± ​SD (n ​= ​3), with *p* ​values determined by two-tailed unpaired *t*-tests, not significant (ns) when *p* ​> ​0.05. SD, standard deviation; HMP, hexametaphosphate.

Rheological experiments provided an important insight into the shear rate response of the formulations; however, the tests performed did not entirely represent the intended delivery device. To provide a more fidelitous assessment of the *in situ* behavior during injection, a displacement controlled compression test was used to obtain a direct quantitative measure. The plunger was compressed at a set rate, and the force required to push the formulation through the needle was measured (Fig. 4A).

Injectability was determined as the ability of the formulation to entirely pass through the needle with a reasonable amount of force. The value at the plateau was taken as the injection force (Fig. 4B, black arrow). Uninjectable systems were defined as those which required a force above 38 ​N to be injected [40]. Formulations in which a precipitate had formed ​and where the solid material did not pass through the needle, had an initial force-displacement trace similar to injectable samples. However, at a certain point (Fig. 4B, light gray arrow), the injection force rapidly increased due to a build-up of precipitate in the syringe, resulting in filter pressing.

A range of parameters, known from the Hagen-Poiseuille equation to determine injectability, were investigated: alginate concentration (corresponding to formulation viscosity), needle diameter, and injection speed [40]. While previous experiments indicated that 0.2 ​M may be the maximum HMP concentration which does not cause phase separation, these additional data may help to identify an optimum alginate concentration in the formulations. Two needle gauges were investigated: the smaller, which is suitable to test the treatment in a rodent model, and the larger which is more appropriate for clinical use. A range of injection speeds were tested; 1 ​mm ​s^−1^ is representative of manual injection [40], however a larger range was tested to examine the effects of shear-thinning.

The required force for injection was found to increase with increasing polymer concentration (Fig. 4C). This was because increasing the alginate concentration increases the formulation viscosity. Doubling the polymer concentration increased the standing viscosity by approximately a factor of 10 (Fig. 2E); however, a more complex relationship was seen for injection force. This is likely due to the shear-thinning behavior of the formulations, which decreased their viscosity as they experienced high shear in the narrow gap of the needle, and the composite geometry in a needle-syringe system.

Decreasing the needle diameter greatly increased the force required for injection (Fig. 4D). This may be attributed to an increase in average flow speed through the smaller diameter needles. Increasing the flow speed increased the injection force (Fig. 4E) because of the higher wall shear stress. It should also be noted that different gauge needles are different lengths, which is another parameter included in the Hagen-Poiseuille equation that is known to affect injection force.

**Fig. 5:**
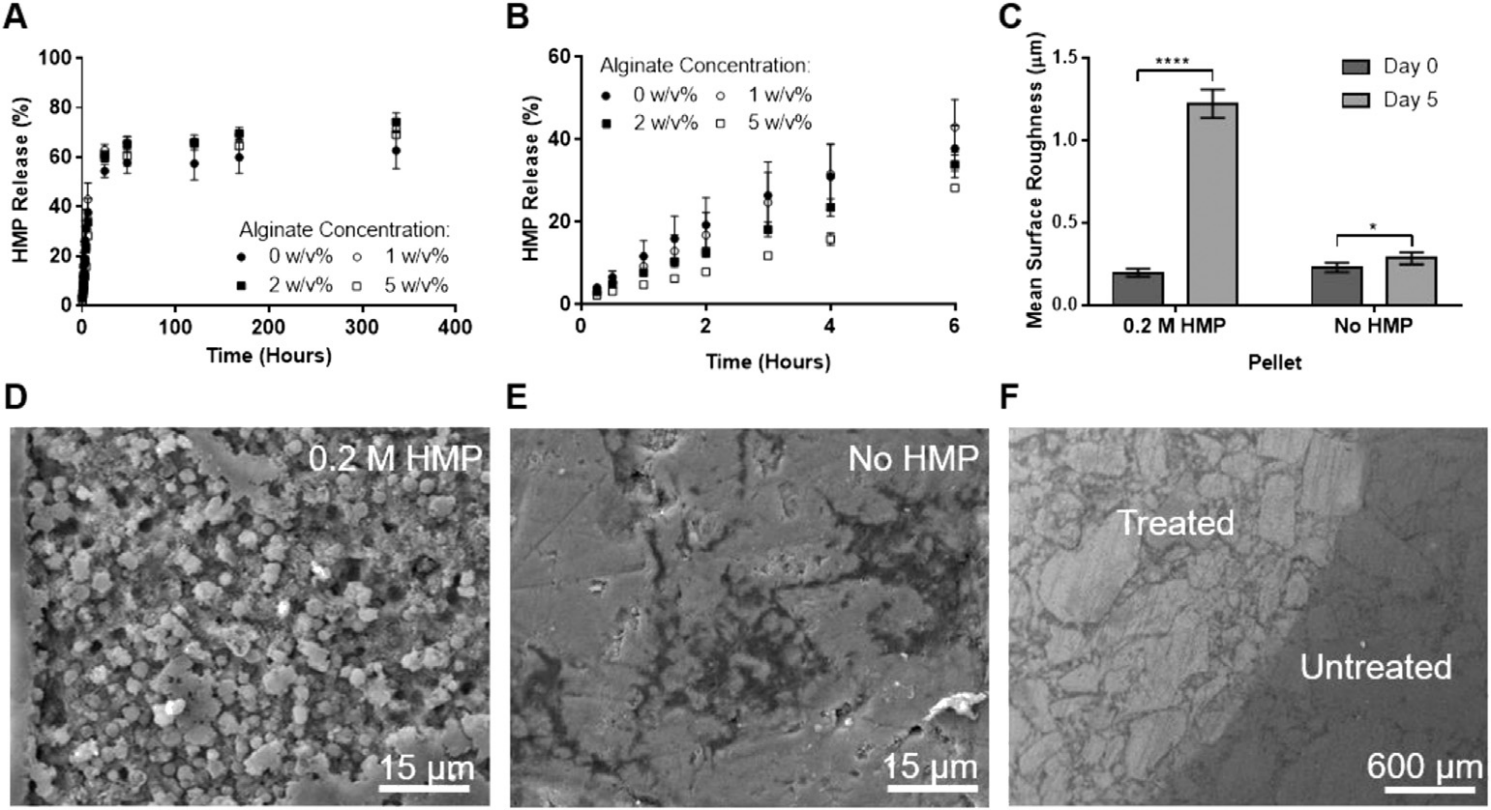
**Formulations release HMP to dissolve HA**. **(A)** Release of HMP over time from formulations (with initial HMP concentration of 0.2 ​M) with varying alginate concentrations. **(B)** Replot of (A), highlighting the release over the first 6 ​h. **(C)** Interferometry data showing the change in surface roughness of a polished HA pellet before and after the application of 2 w/v% alginate with and without 0.2 ​M HMP. Secondary electron SEM images of HA pellets **(D)** after exposure to 0.2 ​M HMP, 2 w/v% alginate formulation, demonstrating roughened topology with rounded particles, **(E)** after exposure to a 2 w/v% alginate solution, and **(F)** boundary between the region treated with a HMP-containing formulation and the untreated area highlighting differences in topography and grain size. (A) and (B) show mean ​± ​SD (n ​= ​3) and (C) shows mean ​± ​SD (n ​= ​6), with *p* ​values determined by two-tailed unpaired *t*-tests, not significant (ns) when *p* ​> ​0.05. SD, standard deviation; HMP, hexametaphosphate; HA, hydroxyapatite; SEM, scanning electron microscopy.

The release of the active component from the formulation over time was assessed *in vitro* through a regular dialysis method. Around 40% of HMP was released in the first 6 ​h, with apparent zero-order kinetics and a further 30% released over the following two weeks (Fig. 5A). From the first release region (Fig. 5B), a higher polymer concentration was found to slow release. Specifically, 2 and 5 w/v% alginate formulations released the HMP 21% and 34% slower, respectively, than the 1 w/v% sample over 6 ​h. This was due to the higher polymer content increasing the path length and retarding solute release.

The demineralizing capacity of the formulations was first tested *in vitro* using sintered HA pellets. Interferometric analysis revealed that applying 0.2 ​M HMP containing formulations to HA increased the surface roughness more than six-fold (Fig. 5C). This was likely because the formulation dissolved the surface of the HA, but the free ions then precipitated out as they cannot flow away in this static *in vitro* system. The observed surface morphology supported this mechanism; the formation of discreet spheres suggested nucleation and growth of solid mineral precipitating from solution (Fig. 5D). A small increase in surface roughness (24%) was also seen for the formulation without HMP. This may be due to some HA dissolution in the aqueous phase, perhaps enhanced by alginate's affinity for calcium, or simply caused by water ingress into the ceramic. In contrast to the active sample, reprecipitation was not observed for the control without HMP (Fig. 5E), and this is likely because supersaturation was not achieved.

The targeted delivery of the formulations is demonstrated by Fig. 5F, where a distinct boundary between regions treated with and without HMP can be seen. Notably, the treated region was lighter in the SEM ​image than the untreated one; this indicates that this area had steeper surfaces. This agrees with the morphology observed at higher magnification (Fig. 5D).

### 3.4 Effect of HMP formulations on biological bone

**Fig. 6:**
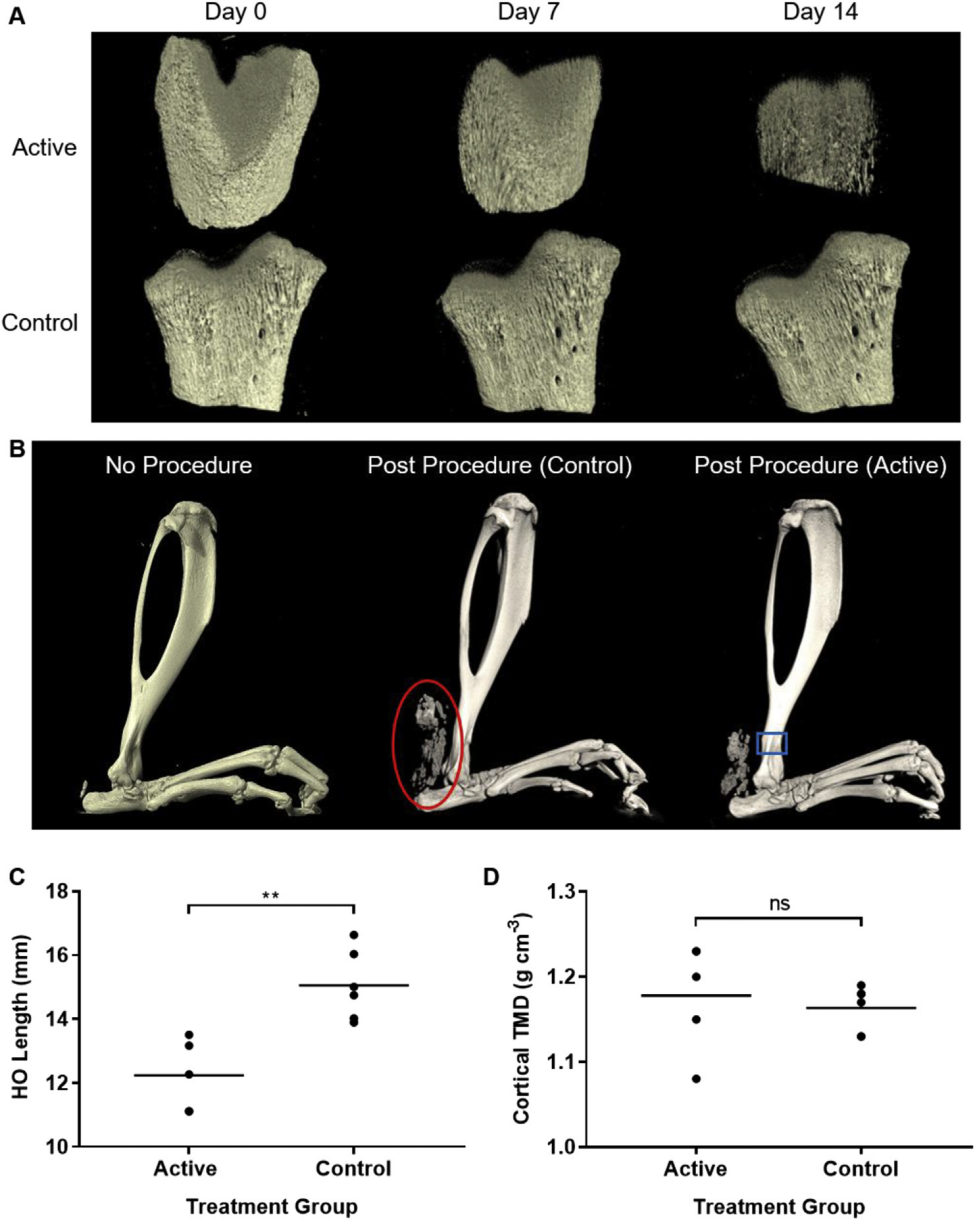
**Formulations demineralise bone *exvivo* and *invivo***. **(A)** Micro-CT reconstructions of distal sections of rat femur, showing the demineralizing capacity of formulations with and without 0.2 ​M HMP. **(B)** Micro-CT reconstructions of the lower hind limbs of rats, which have not (left) ​or have been subjected to Achilles tenotomy and treated with a control (center) or active (right) formulation. The developed HO is shown by the red circle, and the region of the tibia used to calculate TMD is shown by the blue square. **(C)** The length of HO formed in the hind limbs of rats which received prophylactic formulations containing HMP (active) or not (control). **(D)** The TMD of the cortical bone in the tibia, adjacent to the formulation injection site, of rats which received treatment formulations containing HMP (active) or not (control). (C) and (D) show all data points and the mean (n ​= ​5 [active] and n ​= ​6 [control]), with *p* ​values determined by two-tailed unpaired *t*-tests, not significant (ns) when *p* ​> ​0.05. HO, heterotopic ossification; HMP, hexametaphosphate; TMD, tissue mineral density; CT, computed tomography.

Having shown efficacy of the formulations in dissolving HA *in vitro*, the capacity to demineralize the *ex vivo* was studied. These experiments provided further evidence of the potent efficacy of these formulations; mineral volume was reduced by 75% in two weeks (Fig. 6A). The total tissue volume changed little, however, as the collagen matrix of the bone remained.

The promising demineralization efficacy of HMP *in vitro* and *ex vivo* substantiated progression to testing the formulation in an *in vivo* model of HO. Ectopic bone formation was induced by Achilles tenotomy surgery in hind limbs of rats, and the formulations were used to treat the HO formed. Initial model validation studies showed consistency and reliability; 100% of the animals developed HO in the operated (right) limb after 10 weeks (Fig. 6B). No HO was seen in the control (left) limb, and no adverse effects were seen during or after surgery.

The effect of the formulations was then examined, by injecting them into the site of HO formation. An active formulation containing 1 w/v% alginate and 0.2 ​M HMP ​or a control formulation containing 1 w/v% alginate only ​were administered fortnightly. After treatment, the length of HO which formed in the hind limb of the animals was determined via micro-CT. Animals which received the active prophylactic developed, on average, 2.8 ​mm less HO than controls, a reduction of 19% (Fig. 6C, *p* ​< ​0.01). This shows that the formulations are active in a biological system ​and able to significantly reduce the effects of HO in living animals. The TMD of the tibia, adjacent to the site of injection, was also determined ​to assess the effect of the treatment on the surrounding skeletal bone. No significant difference in TMD was observed, which suggested that the active formulation did not demineralize the orthotopic bone (Fig. 6D). This demonstrated that the viscosity recovery (Fig. 2E) and localized targeting (Fig. 5F) seen *in vitro* are maintained *in vivo*.

## 4 Discussion

### 4.1 Shear-thinning formulations with recoverable post-shear viscosity

Current preventions for HO are inconsistent in their efficacy, with a range of undesirable side-effects, and surgical excision has a host of risks. This necessitates the need for the development of a new, minimally invasive prophylactic and treatment ​to improve quality of life for HO sufferers in both military and civilian populations. In this study, we have presented such a treatment, in the form of an injectable formulation. This allows it to be used in a civilian outpatient clinic ​and also in any level of military medical facility; no specialist equipment is required. Furthermore, this treatment is distinct from other proposed prophylaxes, which aim to target the upstream biological pathways of HO. By focusing on the chemical level, this treatment may also be used to dissolve the formed bone, offering a minimally invasive alternative to surgery. This therapy is also favorable from a translational perspective; it is a simple two-component system, and both components are recognized as safe by the United States Food and Drug Administration (FDA) [47,48]. Furthermore, both alginate and HMP are relatively cheap, processing is a single mixing step at ambient temperature, and sterilization can be achieved simply by filtration. These factors lend themselves to a scalable and cost-effective treatment.

The formulations were found to be shear-thinning, as their viscosities decreased with increasing shear rate. This behavior is typical of concentrated polymer solutions, where the chains are entangled. At low shear rates, the chains form new entanglements at the same rate as they are broken by shear, and their viscosity remains constant. At a critical point, the rate of entanglement lags ​and the polymer network breaks down, resulting in a reduction in viscosity [49,50]. The formulations with up to 0.2 ​M HMP are examples of this behavior. Flocculated particles in suspension also display shear-thinning behavior, as the shear forces break apart the flocs, reducing the viscosity. However, these curves have a different shape, with a much steeper shoulder after the Newtonian plateau [51], similar to those seen for the phase-separated samples with >0.2 ​M HMP. Shear-thinning formulations are favorable for injectable systems; their viscosity decreases when they experience the high shear force in the needle during injection ​and thus will take less force to inject than a Newtonian fluid of the same standing viscosity. Shear-thinning would not be possible were the polymers covalently linked ​or linked by strong ionic bonds, as in a calcium alginate gel, such that the bonds between polymers could not be easily broken during injection and reformed *in vivo* [52].

The time taken for the formulations to recover their viscosity, following the high shear force experienced during injection, is also important to this application as short recovery times prevent delocalization. Formulations with 0.2 ​M HMP recovered their viscosity on the removal of shear almost instantaneously. This is expected, as alginate solutions are pseudoplastic materials, whose viscosity is independent of time [53,54]. The formulations with >0.2 ​M HMP, which formed a precipitate, displayed thixotropy. There is time dependence to their shear-thinning behavior, taking around 30 ​s to recover their initial viscosity. This is typical of weakly flocculated particle suspensions; the flocs take a finite amount of time to recoagulate, after the removal of shear [55].

HMP concentration had a profound effect on the rheological properties of the formulations. Low HMP concentrations increased the viscosity of alginate in solution, by increasing interaction density in the entangled polymer network. This is advantageous because it makes the treatment more site-specific, without adding more polymer which the body would be required to eliminate. The peak value of viscosity appeared at around 0.2 ​M HMP, for all alginate concentrations. Further increasing the HMP concentration decreased the viscosity, concurrent with the formation of a precipitate and phase separation. This decrease in viscosity is due to the precipitate layer removing polymer from solution, and thus the formulation moves from an entangled polymer network to a suspended floc system. This interaction behavior is thus the limiting factor for drug loading. In addition to the decrease in viscosity at concentrations exceeding 0.2 ​M, the formation of the precipitate removes the possibility of filter sterilization, which is one of the key advantages of this system. Henceforth, 0.2 ​M HMP systems were investigated for injectability and bioactivity, *in vitro* and *in vivo*.

### 4.2 Interaction between the demineralizing agent and polymer network

The significant effect of HMP concentration on the rheological properties of the formulations suggested there was an interaction, on the molecular level, between the HMP and the polymer. Investigating this via zeta potential measurements suggested that the HMP reduced the electrostatic repulsion between alginate chains, allowing them to come closer together and interact. Increasing salt concentration has previously been shown to increase the zeta potential of alginate solutions [56]. This is because the greater ionic strength reduces the size of the surrounding electrical double layer, allowing for a greater degree of interaction [57]. This effect also increases with increasing ionic valence [58]. The profound effect of HMP on zeta potential may thus be explained by high sodium and hexavalent polyphosphate ion concentrations. The low pKa values of alginate (3.38–3.65, depending on polymer chain composition) and HMP (2.96) ​may explain why pH has a negligible effect on zeta potential [59,60]. Owing to their low pKa values, neither species was substantially protonated in the tested pH range (3.9–10.0), thus the surface charge ​and by extension zeta potential ​did not vary considerably.

The peak shifts in the FTIR spectra suggested that it is the resonating groups on the alginate and HMP which were interacting, and it is proposed that these stabilize around positively charged sodium. There is a high concentration of sodium in these formulations, as both alginate and HMP are sodium salts. Each molecule may form several interactions, as HMP anions have six resonating groups and alginate has one carboxyl group per uronic acid moiety. This interaction increases the viscosity of the formulations, up to 0.2 ​M HMP, which is beneficial for this application; the formulations will have a lower propensity to disperse, without the need for additional polymer. Beyond this limit, the interaction binds the polymers strongly and causes phase separation. This is undesirable from a translational standpoint, as it prevents filter sterilization and may reduce reproducibility of treatment.

### 4.3 Injectable formulations for targeted release

To obtain a more pragmatic understanding of clinical application, quantitative injection tests were performed to measure the force required to inject the formulations through a hypodermic needle. The majority of tests were performed on formulations with 0.2 ​M HMP, as the rheological characterization suggested that this is the optimum drug loading. Tests were also performed with higher HMP concentrations to understand the effect of precipitation on injectability. Injectable samples passed through the needle smoothly in a single stream, which is desirable for clinical application. For uninjectable samples, the force required to extrude the formulation at a clinically relevant flow rate exceeded reasonable limits (38 ​N) [40]. The precipitated samples were injectable until the buildup of solids resulted in filter pressing, which hindered further expulsion through the needle. This behavior is commonly observed in two-phase systems such as cements [61] and further confirms that precipitated formulations are unsuitable for this application.

The force-displacement traces for injectable formulations had two distinct parts (Fig. 4B). First, an initial slope resulting in a peak, which corresponded to compressing the plunger and accelerating the formulation. Second, a plateau region corresponding to the force required to expel the formulation at constant speed [62]. No peak was seen for formulations where the second force was greater than the first. The force required to inject the formulations grew with increasing polymer concentration, increasing injection rate, and decreasing needle size. The force which can sensibly be applied by hand will vary between clinicians; however, a conservative view should be taken so that a given formulation can be delivered by any healthcare professional if required. This limitation will place a threshold on these parameters, particularly the alginate concentration in the formulation, and also the selected needle size.

The study of the *in vitro* release of the HMP from the formulations revealed two phases. In the initial 6 ​h, 40% of the drug was released, and a further 30% was released over the following two weeks. This initial fast release may be due to the system swelling via osmosis; the dialysis tubing swelled to its maximum volume when immersed in the release medium. Once fully swollen, the HMP diffused out of the system, with the polymer chains acting as obstructions to mass transport [63]. This may explain the two distinct release regions. The positive control with no alginate released faster initially ​but the release rate slows earlier than for the other formulations. This may be due to a faster swelling rate, as there is no polymeric hindrance to water ingress. The release *in vivo,* however, may have a different profile. The swelling, while bounded somewhat by the pressure of the surrounding tissue, will be less finitely constricted. Furthermore, the rate of swelling may decrease, as the osmotic pressure in body fluids will be less than that of deionized water, used as the release medium *in vitro*. The profile *in vivo* may therefore be purely swelling controlled ​but over a longer time period. The release of the HMP may be retarded by increasing the alginate concentration ​because the greater network density increases the path length of the HMP. The alginate concentration can therefore be altered to tailor the release profile of HMP; however, this will also affect formulation viscosity and injectability.

The demineralization capacity of the formulation was studied *in vitro* on a pellet of HA. The HMP-containing formulation greatly increased the surface roughness of the pellet, due to dissolution and reprecipitation. This demonstrates the ability of the formulation to dissolve HA, the main mineral constituent of bone; however, *in vivo* reprecipitation is not expected to occur, as the ions can flow away from the site. The formulations also exhibit targeting, with a clear distinction between the treated and untreated area of the pellet. This is promising for *in vivo* studies, as it suggests the formulation will be able to demineralize the HO where it is injected, without damaging the nearby orthotopic bone.

### 4.4 Effect of HMP formulations on biological bone

Having demonstrated that the formulations were injectable ​and dissolved HA *in* *vitro*, the effect of the formulations on biological bone was then studied. This was important in order to understand whether the organic components of bone, such as collagen, prevented demineralization in some way. Femoral condyles were used, as they consist largely of trabecular bone, which has a macroscopic structure more similar to HO than cortical bone. The HMP-containing formulation reduced the mineral volume by over 75% in two weeks ​but did not dissolve the collagen matrix. This is expected, as HMP demineralizes the bone by sequestering calcium, to dissolve the calcium phosphate phase, ​but does not affect the organic phase [38]. This suggests the formulation will not have deleterious effects on the soft tissue surrounding the HO *in vivo*. Although this treatment only removes one component of the HO, it is this mineral component which causes most, if not all, of the complications arising from HO. Demineralizing the ectopic tissue could improve the range of joint motion, prevent skin ulceration, reduce pain, and allow amputees to more easily wear their prostheses.

Having shown efficacy *ex vivo*, the formulations were studied in an animal model of HO. An Achilles tenotomy model was chosen for this study ​because it induces HO reliably from a non-pharmacological event. As the biology of HO formation was not under investigation, blast and injury models, which are more representative but also more harmful and less controlled, were not necessary. Implantation, injection, or genetic modification models, the majority of which induce artificially high bone morphogenetic protein levels, are less representative ​and are no less complex. Initial model validation demonstrated that the animals recovered quickly and that all animals developed HO in the operated limb after 10 weeks. The formulations were then studied using this model, with fortnightly injections following surgery. It should be noted that one animal in the active group died immediately after injection at six weeks. Veterinary review concluded that this was due to unintended intravascular injection, causing an embolism. As a result of this event, future treatments included an aspiration step prior to injection, and no further events were observed.

The length of the developed HO was chosen as the marker of efficacy ​because it is the length of the spurs of bone that is responsible for the chronic pain, skin ulceration, and ankylosis which may be experienced by patients. These spurs may thus be a promising initial target for this type of treatment ​to alleviate these symptoms. The length of HO was found to be significantly reduced by the active formulation, which suggested that the proposed therapeutic may help to relieve these symptoms ​and be an efficacious prophylactic or treatment for formed HO.

There was no significant change in the TMD of the tibia, adjacent to the injection site. In addition to targeted delivery to the HO site, this may also be because HMP is more effective in low pH conditions, which exist in the local inflammatory site of HO [38]. Furthermore, as treatment took place during HO formation, HMP may be more effective at preventing mineralization than demineralizing the existing skeletal bone. Formed HO has a floral appearance ​and thus has a much larger specific surface area than the skeletal bone for dissolution and reaction of the mineral phase. Moreover, mature skeletal bone is covered by the periosteum, which may offer some protection against HMP. These factors may all contribute to the preferential demineralization of HO over skeletal bone.

This study demonstrates that targeted demineralization may be a viable strategy to prevent or treat HO. However, this preliminary study is limited to the examination of one delivery vehicle ​and treatment strategy *in vivo*. While colloidal alginate is a suitable delivery vehicle, other systems may provide improved rheological or release properties ​and sustain HMP release over a longer period. Further *in vitro* and *ex vivo* experiments in physiologically representative environments, followed by *in vivo* studies, to determine the material and release properties of the formulations over time are also warranted. In addition, this study examines a single dosing regimen. HMP concentration, dosing timing, and frequency are all factors that can be optimized, which may give increased demineralization *in vivo*. The effect of applying HMP to HO once completely formed, ​and on the HO once treatment has ceased, ​are also important considerations. Other effects of injected HMP, such as temporal changes to local and systemic calcium concentration, may also help to elucidate the precise mechanism of HO demineralization *in vivo*.

## 5 Conclusion

This study has shown that HMP, a potent calcium chelator, can be incorporated into a vehicle with optimal material properties for injection and targeted delivery. This formulation is effective at dissolving HA, demineralizing biological bone, and can curb the growth of HO in an animal model, without affecting the adjacent skeletal bone. These data suggest ​that demineralization may be a viable strategy for HO management and, unlike biological prophylaxes, could act as an alternative to surgery. This preliminary study warrants further exploration into this area ​to provide new therapies and improve the quality of life for sufferers of HO and other pathological ossification and calcification diseases.

## CRediT authorship contribution statement

**T.E. Robinson:** Conceptualization, Methodology, Formal analysis, Investigation, Writing - original draft, Writing - review & editing, Visualization, Funding acquisition. **N.M. Eisenstein:** Conceptualization, Methodology, Formal analysis, Investigation, Writing - review & editing, Funding acquisition. **S.C. Cox:** Conceptualization, Writing - review & editing, Supervision. **R.J.A. Moakes:** Conceptualization, Writing - review & editing. **A.M. Thompson:** Investigation. **Z. Ahmed:** Methodology, Investigation, Writing - review & editing. **E.A.B. Hughes:** Methodology, Writing - review & editing. **L.J. Hill:** Methodology, Writing - review & editing. **S.A. Stapley:** Conceptualization, Writing - review & editing, Supervision, Funding acquisition. **L.M. Grover:** Conceptualization, Writing - review & editing, Supervision, Funding acquisition.

## Declaration of competing interest

The authors have no competing interests to declare.
